# *catena*-Poly[[bis­(μ-3-carboxy­benzoato)bis­(1,10-phenanthroline)tricopper(II)]-di-μ_3_-isophthalato]

**DOI:** 10.1107/S1600536808036908

**Published:** 2008-11-20

**Authors:** Yan An, Xiao-Feng Li, Li-Hua Dong, Yan-Sheng Yin

**Affiliations:** a Institute of Marine Materials Science and Engineering, Shanghai Maritime University, Shanghai 200135, People’s Republic of China

## Abstract

The title copper coordination polymer, [Cu_3_(C_8_H_4_O_4_)_2_(C_8_H_5_O_4_)_2_(C_10_H_8_N_2_)_2_]_*n*_, was synthesized by reacting Cu(NO_3_)_2_, isophthalic acid and 1,10-phenanthroline under hydro­thermal conditions. The trinuclear unit presents a central almost planar CuO_4_ chromophore with the cation on a symmetry center, and two symmetry-related CuN_2_O_3_ groups with the metal centre in a distorted square-pyramidal environment. These units are bridged by isophthalate ligands into one-dimensional double-chain coordination polymers which are, in turn, connected by various π–π stacking inter­actions (face-to-face distance *ca* 3.45 Å) and O—H⋯O hydrogen bonds, forming a three-dimensional supra­molecular network.

## Related literature

For related literature on the design and construction of coordination polymers, see: Amabilino & Stoddart (1995 ▶); Han *et al.* (2005 ▶, 2007 ▶, 2008 ▶); He & Han (2007 ▶); Ma *et al.* (2007 ▶).
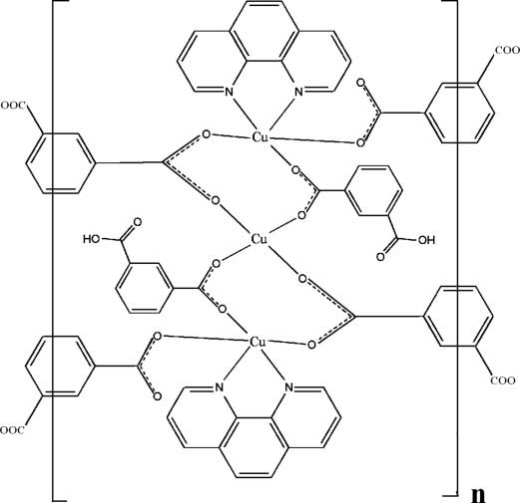


## Experimental

### 

#### Crystal data

[Cu_3_(C_8_H_4_O_4_)_2_(C_8_H_5_O_4_)_2_(C_10_H_8_N_2_)_2_]
*M* *_r_* = 1209.5
Triclinic, 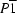

*a* = 10.383 (1) Å
*b* = 10.659 (1) Å
*c* = 11.754 (1) Å
α = 83.147 (1)°
β = 86.191 (1)°
γ = 71.134 (1)°
*V* = 1221.6 (2) Å^3^
*Z* = 1
Mo *K*α radiation
μ = 1.38 mm^−1^
*T* = 293 (2) K
0.37 × 0.32 × 0.23 mm

#### Data collection

Bruker SMART APEX area-detector diffractometer
Absorption correction: multi-scan (*SADABS*; Sheldrick, 1996 ▶) *T*_min_ = 0.631, *T*_max_ = 0.738
9575 measured reflections
4741 independent reflections
4241 reflections with *I* > 2σ(*I*)
*R*_int_ = 0.063

#### Refinement

*R*[*F* ^2^ > 2σ(*F* ^2^)] = 0.031
*wR*(*F* ^2^) = 0.089
*S* = 1.09
4741 reflections
358 parameters
H-atom parameters constrained
Δρ_max_ = 0.43 e Å^−3^
Δρ_min_ = −0.35 e Å^−3^

### 

Data collection: *SMART* (Bruker, 2001 ▶); cell refinement: *SAINT* (Bruker, 2001 ▶); data reduction: *SAINT*; program(s) used to solve structure: *SHELXS97* (Sheldrick, 2008 ▶); program(s) used to refine structure: *SHELXL97* (Sheldrick, 2008 ▶); molecular graphics: *SHELXTL* (Sheldrick, 2008 ▶); software used to prepare material for publication: *SHELXTL*.

## Supplementary Material

Crystal structure: contains datablocks I, global. DOI: 10.1107/S1600536808036908/bg2210sup1.cif

Structure factors: contains datablocks I. DOI: 10.1107/S1600536808036908/bg2210Isup2.hkl

Additional supplementary materials:  crystallographic information; 3D view; checkCIF report

## Figures and Tables

**Table:** 

Cu1—O6	1.923 (1)
Cu1—O2	2.010 (1)
Cu2—O3^i^	1.935 (1)
Cu2—O1	1.951 (1)
Cu2—N2	2.008 (2)
Cu2—N1	2.014 (2)
Cu2—O5	2.278 (1)

**Table:** 

O6—Cu1—O2^ii^	92.30 (6)
O6—Cu1—O2	87.70 (6)
O3^i^—Cu2—O1	92.66 (6)
O1—Cu2—N2	168.29 (7)
N2—Cu2—N1	81.94 (7)
O1—Cu2—O5	91.79 (6)
N1—Cu2—O5	98.46 (6)

Symmetry codes: (i) 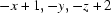; (ii) 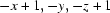.

**Table 2:** Hydrogen-bond geometry (Å, °)

*D*—H⋯*A*	*D*—H	H⋯*A*	*D*⋯*A*	*D*—H⋯*A*
O7—H7*A*⋯O4^iii^	0.82	1.73	2.539 (2)	170

Symmetry code: (iii) 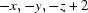.

**Fig. 1.**
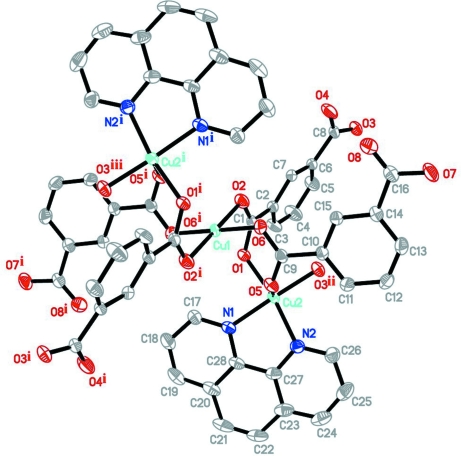
A trinuclear [Cu3(ip)2(Hip)2(phen)2] subunit with thermal ellipsoids at 30% probability. Symmetry transformations used to generate equivalent atoms: (i) -x+1,-y,-z+1; (ii) -x+1,-y,-z+2; (iii) x,y,z-1.

**Fig. 2.**
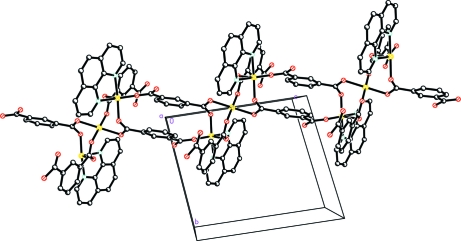
Packing view of I drawn along c and depicting the double chain fragment.

## supplementary crystallographic information

### Comment

The design and construction of coordination polymers has attracted much attention owing to their intriguing topologies and potential applications as functional materials (Han *et al.*, 2008, Ma *et al.*, 2007). Many networks with various structural motifs have been documented in the past decade (Amabilino *et al.*, 1995). Unlike pyridine 2,4-, 3,4- 2,5- and 2,6-dicarboxylic acids which were widely reported as bridging ligands to assemble various coordination polymers, isophthalic acid (H_2_ip) has been reported only scarcely in the role of multicarboxylate ligand (Han *et al.*, 2007). We report here the synthesis and structure of the title copper(II) coordination polymer [Cu_3_(C_8_H_4_O_4_)_2_(C_8_H_5_O_4_)_2_(C_10_H_8_N_2_)]_n_,assembled from isophthalic acid, 1,10-phenanthroline(phen) and copper(II), (I).

In I, there is one and a half Cu(II) ions (Cu1 lies on center of symmetry), one ip, one Hip and one phen ligand in each independent crystallographic unit. Each Cu2 ion is coordinated by three oxygen atoms from two ip ligands and one Hip ligand in a mono-bidentate and bidentate coordination modes and two nitrogen atoms from a chelate phen ligand to furnish a distorted square pyramidal geometry. On the other hand, each Cu1 atom is four-coordinated by four oxygen atoms, forming a slightly distorted square geometry. (Fig.1 and Table 1).

The carboxylate oxygen atoms bridge three copper atoms (Cu2, Cu1 and Cu2^i^) *via* the *syn*–anti O,*O*-bridges to form a trinuclear [Cu_3_(ip)_2_(Hip)_2_(phen)_2_] subunit (Fig. 1), which are interconnected through the bridging ip groups to form an infinite one-dimensional double chain with Cu···Cu distances of 3.755 (3) and 9.994 (3) Å (Fig. 2). The lateral phen ligands from adjacent double-chains are paired to furnish moderately strong π—π stacking interactions (face-face distance *ca* 3.45 (1) Å) (He *et al.*,2007, Han *et al.*, 2005), which extend the double-chains into two-dimensional wavelike layers parallel to the *ab* plane in the lattice. These layers are further linked *via* strong hydrogen bonds between uncoordinated carboxylate oxygen atoms of ip ligands (Table 2), forming a three-dimensional supramolecular network.

### Experimental

A mixture of Cu(NO_3_)_2_.2H_2_O (0.5 mmol, 0.120 g), isophthalic acid (0.5 mmol, 0.084 g), NaOH (1 mmol, 0.04 g), and water (10 ml) was mixed in a 23 ml Teflon reactor, which was heated at 453 K for six days and then cooled to room temperature at a rate of 5 K h^-1^. Yield: 48%. CH&N analysis for C_20_H_17_N_2_O_8_Cu_1.5_ (found/calc): C, 47.05(47.22), H, 2.64(3.37), N, 5.73%(5.51%).

### Refinement

The H atoms were placed at calculated positions in the riding model approximation (C—H 0.93 Å,*O*—H 0.82 Å), with their temperature factors set to 1.2 times those of the equivalent isotropic temperature factors of the parent atoms.

### Crystal data

**Table:**

[Cu_3_(C_8_H_4_O_4_)_2_(C_8_H_5_O_4_)_2_(C_10_H_8_N_2_)_2_]	*Z* = 1
*M**_r_* = 1209.5	*F*_000_ = 613
Triclinic, *P*1	*D*_x_ = 1.644 Mg m^−^^3^
Hall symbol: -P 1	Mo *K*α radiation λ = 0.71073 Å
*a* = 10.383 (1) Å	Cell parameters from 2356 reflections
*b* = 10.659 (1) Å	θ = 2.3–25.0º
*c* = 11.754 (1) Å	µ = 1.38 mm^−^^1^
α = 83.147 (1)º	*T* = 293 (2) K
β = 86.191 (1)º	Block, green
γ = 71.134 (1)º	0.37 × 0.32 × 0.23 mm
*V* = 1221.6 (2) Å^3^	

### Data collection

**Table:**

Bruker SMART APEX area-detector diffractometer	4741 independent reflections
Radiation source: fine-focus sealed tube	4241 reflections with *I* > 2σ(*I*)
Monochromator: graphite	*R*_int_ = 0.063
*T* = 293(2) K	θ_max_ = 26.0º
φ and ω scans	θ_min_ = 2.0º
Absorption correction: multi-scan(SADABS; Sheldrick, 1996)	*h* = −12→12
*T*_min_ = 0.631, *T*_max_ = 0.738	*k* = −13→13
9575 measured reflections	*l* = −12→14

### Refinement

**Table:**

Refinement on *F*^2^	Secondary atom site location: difference Fourier map
Least-squares matrix: full	Hydrogen site location: inferred from neighbouring sites
*R*[*F*^2^ > 2σ(*F*^2^)] = 0.031	H-atom parameters constrained
*wR*(*F*^2^) = 0.089	*w* = 1/[σ^2^(*F*_o_^2^) + (0.0453*P*)^2^ + 0.1215*P*] where *P* = (*F*_o_^2^ + 2*F*_c_^2^)/3
*S* = 1.09	(Δ/σ)_max_ = 0.001
4741 reflections	Δρ_max_ = 0.43 e Å^−^^3^
358 parameters	Δρ_min_ = −0.35 e Å^−^^3^
Primary atom site location: structure-invariant direct methods	Extinction correction: none

### Special details

**Table:**

Geometry. All e.s.d.'s (except the e.s.d. in the dihedral angle between two l.s. planes) are estimated using the full covariance matrix. The cell e.s.d.'s are taken into account individually in the estimation of e.s.d.'s in distances, angles and torsion angles; correlations between e.s.d.'s in cell parameters are only used when they are defined by crystal symmetry. An approximate (isotropic) treatment of cell e.s.d.'s is used for estimating e.s.d.'s involving l.s. planes.
Refinement. Refinement of *F*^2^ against ALL reflections. The weighted *R*-factor *wR* and goodness of fit *S* are based on *F*^2^, conventional *R*-factors *R* are based on *F*, with *F* set to zero for negative *F*^2^. The threshold expression of *F*^2^ > σ(*F*^2^) is used only for calculating *R*-factors(gt) *etc*. and is not relevant to the choice of reflections for refinement. *R*-factors based on *F*^2^ are statistically about twice as large as those based on *F*, and *R*- factors based on ALL data will be even larger.

### Fractional atomic coordinates and isotropic or equivalent isotropic displacement parameters (Å^2^)

**Table:**

	*x*	*y*	*z*	*U*_iso_*/*U*_eq_	
Cu1	0.5000	0.0000	0.5000	0.02502 (10)	
Cu2	0.76902 (2)	−0.22278 (2)	0.697826 (19)	0.03033 (10)	
C1	0.57791 (19)	0.04808 (18)	0.70129 (16)	0.0290 (4)	
C2	0.57869 (19)	0.09529 (18)	0.81581 (16)	0.0281 (4)	
C3	0.7018 (2)	0.0843 (2)	0.86308 (19)	0.0404 (5)	
H3	0.7831	0.0514	0.8219	0.048*	
C4	0.7030 (2)	0.1222 (3)	0.9707 (2)	0.0493 (6)	
H4	0.7852	0.1171	1.0012	0.059*	
C5	0.5830 (2)	0.1680 (2)	1.03389 (18)	0.0402 (5)	
H5	0.5850	0.1920	1.1071	0.048*	
C6	0.46010 (18)	0.17821 (17)	0.98864 (16)	0.0278 (4)	
C7	0.45796 (18)	0.14417 (17)	0.87873 (16)	0.0268 (4)	
H7	0.3752	0.1541	0.8468	0.032*	
C8	0.3300 (2)	0.22144 (19)	1.06009 (16)	0.0316 (4)	
C9	0.47836 (18)	−0.22297 (18)	0.61761 (15)	0.0280 (4)	
C10	0.41202 (18)	−0.30677 (18)	0.69616 (16)	0.0282 (4)	
C11	0.4890 (2)	−0.4306 (2)	0.74662 (19)	0.0376 (5)	
H11	0.5811	−0.4645	0.7271	0.045*	
C12	0.4301 (2)	−0.5038 (2)	0.8255 (2)	0.0470 (6)	
H12	0.4824	−0.5871	0.8582	0.056*	
C13	0.2936 (2)	−0.4538 (2)	0.8561 (2)	0.0419 (5)	
H13	0.2541	−0.5030	0.9096	0.050*	
C14	0.21559 (19)	−0.32961 (19)	0.80667 (16)	0.0315 (4)	
C15	0.27491 (18)	−0.25778 (19)	0.72611 (16)	0.0287 (4)	
H15	0.2221	−0.1757	0.6917	0.034*	
C16	0.0685 (2)	−0.2698 (2)	0.83842 (17)	0.0358 (4)	
C17	0.9058 (2)	−0.1207 (2)	0.48733 (18)	0.0392 (5)	
H17	0.8308	−0.0438	0.4813	0.047*	
C18	1.0192 (2)	−0.1282 (3)	0.4132 (2)	0.0484 (6)	
H18	1.0188	−0.0570	0.3590	0.058*	
C19	1.1296 (2)	−0.2400 (3)	0.4213 (2)	0.0512 (6)	
H19	1.2060	−0.2444	0.3739	0.061*	
C20	1.1287 (2)	−0.3489 (2)	0.5008 (2)	0.0435 (5)	
C21	1.2363 (2)	−0.4740 (3)	0.5150 (2)	0.0554 (7)	
H21	1.3160	−0.4853	0.4705	0.067*	
C22	1.2253 (2)	−0.5748 (3)	0.5903 (2)	0.0536 (7)	
H22	1.2964	−0.6550	0.5957	0.064*	
C23	1.1066 (2)	−0.5616 (2)	0.6627 (2)	0.0451 (6)	
C24	1.0861 (3)	−0.6618 (2)	0.7445 (2)	0.0533 (6)	
H24	1.1520	−0.7454	0.7528	0.064*	
C25	0.9713 (3)	−0.6371 (2)	0.8110 (2)	0.0554 (7)	
H25	0.9574	−0.7037	0.8643	0.066*	
C26	0.8730 (3)	−0.5104 (2)	0.7994 (2)	0.0435 (5)	
H26	0.7956	−0.4937	0.8471	0.052*	
C27	1.00094 (19)	−0.4393 (2)	0.65414 (18)	0.0348 (4)	
C28	1.01068 (19)	−0.3337 (2)	0.57116 (17)	0.0334 (4)	
N1	0.90243 (16)	−0.22039 (16)	0.56575 (14)	0.0318 (4)	
N2	0.88699 (17)	−0.41398 (17)	0.72270 (14)	0.0345 (4)	
O1	0.68897 (14)	−0.03047 (14)	0.66264 (12)	0.0370 (3)	
O2	0.47142 (14)	0.08815 (14)	0.64570 (11)	0.0363 (3)	
O3	0.34517 (15)	0.22533 (15)	1.16551 (12)	0.0386 (3)	
O4	0.22068 (15)	0.25058 (19)	1.01149 (13)	0.0511 (4)	
O5	0.60479 (13)	−0.26213 (14)	0.60173 (12)	0.0346 (3)	
O6	0.40038 (13)	−0.11313 (13)	0.57278 (12)	0.0352 (3)	
O7	0.02655 (15)	−0.34182 (18)	0.92198 (14)	0.0495 (4)	
H7A	−0.0540	−0.3045	0.9375	0.074*	
O8	−0.00380 (15)	−0.16831 (17)	0.78902 (15)	0.0524 (4)	

### Atomic displacement parameters (Å^2^)

**Table:**

	*U*^11^	*U*^22^	*U*^33^	*U*^12^	*U*^13^	*U*^23^
Cu1	0.02711 (17)	0.02716 (17)	0.01884 (17)	−0.00744 (13)	0.00295 (12)	−0.00007 (12)
Cu2	0.03051 (15)	0.03137 (15)	0.02253 (14)	−0.00187 (10)	0.00550 (10)	−0.00313 (10)
C1	0.0350 (10)	0.0269 (9)	0.0239 (9)	−0.0098 (8)	0.0072 (8)	−0.0022 (7)
C2	0.0332 (10)	0.0256 (9)	0.0233 (9)	−0.0073 (7)	0.0041 (7)	−0.0027 (7)
C3	0.0300 (10)	0.0534 (13)	0.0360 (11)	−0.0096 (9)	0.0087 (8)	−0.0137 (10)
C4	0.0313 (11)	0.0773 (17)	0.0428 (13)	−0.0178 (11)	0.0010 (9)	−0.0201 (12)
C5	0.0427 (12)	0.0531 (13)	0.0272 (11)	−0.0156 (10)	0.0017 (9)	−0.0143 (9)
C6	0.0324 (9)	0.0240 (8)	0.0244 (9)	−0.0072 (7)	0.0063 (7)	−0.0019 (7)
C7	0.0287 (9)	0.0254 (9)	0.0236 (9)	−0.0064 (7)	0.0017 (7)	0.0000 (7)
C8	0.0367 (10)	0.0292 (9)	0.0256 (10)	−0.0084 (8)	0.0083 (8)	−0.0014 (8)
C9	0.0328 (10)	0.0300 (9)	0.0229 (9)	−0.0120 (8)	0.0034 (7)	−0.0057 (7)
C10	0.0307 (9)	0.0292 (9)	0.0261 (9)	−0.0118 (8)	0.0020 (7)	−0.0035 (7)
C11	0.0293 (10)	0.0335 (10)	0.0447 (12)	−0.0058 (8)	0.0044 (9)	0.0019 (9)
C12	0.0408 (12)	0.0332 (11)	0.0579 (15)	−0.0059 (9)	0.0016 (10)	0.0124 (10)
C13	0.0416 (11)	0.0403 (11)	0.0437 (13)	−0.0181 (9)	0.0045 (10)	0.0073 (9)
C14	0.0315 (10)	0.0371 (10)	0.0286 (10)	−0.0148 (8)	0.0012 (8)	−0.0042 (8)
C15	0.0295 (9)	0.0299 (9)	0.0258 (9)	−0.0087 (7)	0.0001 (7)	−0.0016 (7)
C16	0.0318 (10)	0.0483 (12)	0.0290 (10)	−0.0154 (9)	0.0015 (8)	−0.0050 (9)
C17	0.0418 (11)	0.0409 (11)	0.0333 (11)	−0.0121 (9)	0.0054 (9)	−0.0052 (9)
C18	0.0521 (14)	0.0549 (14)	0.0419 (13)	−0.0237 (11)	0.0148 (11)	−0.0100 (11)
C19	0.0429 (13)	0.0716 (17)	0.0460 (14)	−0.0258 (12)	0.0184 (11)	−0.0227 (12)
C20	0.0322 (11)	0.0585 (14)	0.0417 (12)	−0.0116 (10)	0.0052 (9)	−0.0240 (11)
C21	0.0298 (11)	0.0708 (17)	0.0608 (16)	−0.0020 (11)	0.0083 (11)	−0.0329 (14)
C22	0.0359 (12)	0.0541 (15)	0.0596 (16)	0.0092 (11)	−0.0062 (11)	−0.0264 (13)
C23	0.0415 (12)	0.0417 (12)	0.0465 (13)	0.0019 (10)	−0.0133 (10)	−0.0183 (10)
C24	0.0591 (15)	0.0371 (12)	0.0533 (15)	0.0052 (11)	−0.0219 (13)	−0.0109 (11)
C25	0.0806 (19)	0.0386 (12)	0.0422 (14)	−0.0110 (12)	−0.0198 (13)	0.0021 (10)
C26	0.0534 (13)	0.0417 (12)	0.0312 (11)	−0.0092 (10)	−0.0035 (10)	−0.0026 (9)
C27	0.0295 (10)	0.0385 (11)	0.0329 (11)	−0.0020 (8)	−0.0058 (8)	−0.0124 (8)
C28	0.0256 (9)	0.0413 (11)	0.0325 (10)	−0.0056 (8)	0.0001 (8)	−0.0151 (9)
N1	0.0282 (8)	0.0355 (9)	0.0305 (9)	−0.0069 (7)	0.0037 (7)	−0.0099 (7)
N2	0.0363 (9)	0.0350 (9)	0.0285 (9)	−0.0054 (7)	−0.0022 (7)	−0.0052 (7)
O1	0.0430 (8)	0.0321 (7)	0.0273 (7)	−0.0019 (6)	0.0116 (6)	−0.0055 (6)
O2	0.0366 (7)	0.0459 (8)	0.0253 (7)	−0.0103 (6)	0.0030 (6)	−0.0096 (6)
O3	0.0442 (8)	0.0455 (8)	0.0239 (7)	−0.0119 (7)	0.0103 (6)	−0.0078 (6)
O4	0.0315 (8)	0.0806 (12)	0.0331 (8)	−0.0077 (8)	0.0078 (7)	−0.0072 (8)
O5	0.0279 (7)	0.0439 (8)	0.0324 (8)	−0.0122 (6)	0.0072 (6)	−0.0075 (6)
O6	0.0332 (7)	0.0342 (7)	0.0350 (8)	−0.0104 (6)	0.0052 (6)	0.0047 (6)
O7	0.0308 (7)	0.0736 (11)	0.0401 (9)	−0.0171 (7)	0.0085 (6)	0.0055 (8)
O8	0.0360 (8)	0.0567 (10)	0.0538 (10)	−0.0048 (7)	0.0072 (8)	0.0032 (8)

### Geometric parameters (Å, °)

**Table:**

Cu1—O6	1.923 (1)	C13—C14	1.390 (3)
Cu1—O2^i^	2.010 (1)	C13—H13	0.93
Cu1—O2	2.010 (1)	C14—C15	1.384 (3)
Cu2—O3^ii^	1.935 (1)	C14—C16	1.493 (3)
Cu2—O1	1.951 (1)	C15—H15	0.93
Cu2—N2	2.008 (2)	C16—O8	1.205 (3)
Cu2—N1	2.014 (2)	C16—O7	1.313 (3)
Cu2—O5	2.278 (1)	C17—N1	1.328 (3)
C1—O2	1.247 (2)	C17—C18	1.404 (3)
C1—O1	1.278 (2)	C17—H17	0.93
C1—C2	1.494 (3)	C18—C19	1.360 (3)
C2—C7	1.391 (2)	C18—H18	0.93
C2—C3	1.392 (3)	C19—C20	1.403 (4)
C3—C4	1.375 (3)	C19—H19	0.93
C3—H3	0.93	C20—C28	1.407 (3)
C4—C5	1.383 (3)	C20—C21	1.436 (3)
C4—H4	0.9300	C21—C22	1.337 (4)
C5—C6	1.382 (3)	C21—H21	0.93
C5—H5	0.93	C22—C23	1.429 (4)
C6—C7	1.386 (3)	C22—H22	0.93
C6—C8	1.510 (2)	C23—C27	1.404 (3)
C7—H7	0.93	C23—C24	1.409 (4)
C8—O4	1.236 (2)	C24—C25	1.351 (4)
C8—O3	1.266 (2)	C24—H24	0.93
C9—O5	1.251 (2)	C25—C26	1.402 (3)
C9—O6	1.265 (2)	C25—H25	0.93
C9—C10	1.497 (3)	C26—N2	1.322 (3)
C10—C15	1.386 (2)	C26—H26	0.93
C10—C11	1.388 (3)	C27—N2	1.357 (3)
C11—C12	1.380 (3)	C27—C28	1.424 (3)
C11—H11	0.9300	C28—N1	1.356 (2)
C12—C13	1.383 (3)	O3—Cu2^ii^	1.9349 (14)
C12—H12	0.9300	O7—H7A	0.82
			
O6—Cu1—O2^i^	92.30 (6)	C14—C15—C10	120.78 (17)
O6^i^—Cu1—O2	92.30 (6)	C14—C15—H15	119.6
O6—Cu1—O2	87.70 (6)	C10—C15—H15	119.6
O3^ii^—Cu2—O1	92.66 (6)	O8—C16—O7	124.33 (19)
O3^ii^—Cu2—N2	96.52 (7)	O8—C16—C14	122.77 (19)
O1—Cu2—N2	168.29 (7)	O7—C16—C14	112.85 (18)
O3^ii^—Cu2—N1	174.37 (6)	N1—C17—C18	122.0 (2)
O1—Cu2—N1	88.24 (6)	N1—C17—H17	119.0
N2—Cu2—N1	81.94 (7)	C18—C17—H17	119.0
O3^ii^—Cu2—O5	87.08 (6)	C19—C18—C17	119.6 (2)
O1—Cu2—O5	91.79 (6)	C19—C18—H18	120.2
N2—Cu2—O5	95.92 (6)	C17—C18—H18	120.2
N1—Cu2—O5	98.46 (6)	C18—C19—C20	120.2 (2)
O2—C1—O1	122.29 (17)	C18—C19—H19	119.9
O2—C1—C2	119.67 (16)	C20—C19—H19	119.9
O1—C1—C2	118.02 (17)	C19—C20—C28	116.6 (2)
C7—C2—C3	119.32 (17)	C19—C20—C21	125.7 (2)
C7—C2—C1	120.71 (17)	C28—C20—C21	117.8 (2)
C3—C2—C1	119.89 (17)	C22—C21—C20	121.9 (2)
C4—C3—C2	119.93 (18)	C22—C21—H21	119.0
C4—C3—H3	120.0	C20—C21—H21	119.0
C2—C3—H3	120.0	C21—C22—C23	121.3 (2)
C3—C4—C5	120.5 (2)	C21—C22—H22	119.3
C3—C4—H4	119.7	C23—C22—H22	119.3
C5—C4—H4	119.7	C27—C23—C24	116.2 (2)
C6—C5—C4	120.24 (19)	C27—C23—C22	118.5 (2)
C6—C5—H5	119.9	C24—C23—C22	125.3 (2)
C4—C5—H5	119.9	C25—C24—C23	120.3 (2)
C5—C6—C7	119.43 (17)	C25—C24—H24	119.8
C5—C6—C8	120.12 (17)	C23—C24—H24	119.8
C7—C6—C8	120.41 (17)	C24—C25—C26	119.6 (2)
C6—C7—C2	120.50 (18)	C24—C25—H25	120.2
C6—C7—H7	119.7	C26—C25—H25	120.2
C2—C7—H7	119.7	N2—C26—C25	122.1 (2)
O4—C8—O3	126.40 (18)	N2—C26—H26	118.9
O4—C8—C6	118.12 (17)	C25—C26—H26	118.9
O3—C8—C6	115.48 (18)	N2—C27—C23	123.3 (2)
O5—C9—O6	123.92 (17)	N2—C27—C28	116.57 (17)
O5—C9—C10	119.67 (17)	C23—C27—C28	120.1 (2)
O6—C9—C10	116.40 (15)	N1—C28—C20	123.2 (2)
C15—C10—C11	118.97 (17)	N1—C28—C27	116.50 (17)
C15—C10—C9	120.43 (16)	C20—C28—C27	120.31 (19)
C11—C10—C9	120.43 (16)	C17—N1—C28	118.42 (17)
C12—C11—C10	120.60 (18)	C17—N1—Cu2	129.29 (14)
C12—C11—H11	119.7	C28—N1—Cu2	111.69 (14)
C10—C11—H11	119.7	C26—N2—C27	118.37 (18)
C11—C12—C13	120.21 (19)	C26—N2—Cu2	129.40 (15)
C11—C12—H12	119.9	C27—N2—Cu2	112.03 (14)
C13—C12—H12	119.9	C1—O1—Cu2	129.14 (13)
C12—C13—C14	119.75 (19)	C1—O2—Cu1	110.19 (11)
C12—C13—H13	120.1	C8—O3—Cu2^ii^	134.22 (14)
C14—C13—H13	120.1	C9—O5—Cu2	129.50 (12)
C15—C14—C13	119.68 (18)	C9—O6—Cu1	112.10 (11)
C15—C14—C16	118.41 (18)	C16—O7—H7A	109.5
C13—C14—C16	121.91 (18)		
			
O2—C1—C2—C7	22.0 (3)	C19—C20—C28—C27	179.90 (19)
O1—C1—C2—C7	−159.56 (17)	C21—C20—C28—C27	0.7 (3)
O2—C1—C2—C3	−161.19 (19)	N2—C27—C28—N1	−2.8 (3)
O1—C1—C2—C3	17.2 (3)	C23—C27—C28—N1	177.86 (17)
C7—C2—C3—C4	−0.3 (3)	N2—C27—C28—C20	176.54 (18)
C1—C2—C3—C4	−177.1 (2)	C23—C27—C28—C20	−2.8 (3)
C2—C3—C4—C5	1.7 (4)	C18—C17—N1—C28	−2.1 (3)
C3—C4—C5—C6	−1.1 (4)	C18—C17—N1—Cu2	168.21 (16)
C4—C5—C6—C7	−1.0 (3)	C20—C28—N1—C17	2.6 (3)
C4—C5—C6—C8	176.8 (2)	C27—C28—N1—C17	−178.15 (18)
C5—C6—C7—C2	2.4 (3)	C20—C28—N1—Cu2	−169.34 (16)
C8—C6—C7—C2	−175.41 (16)	C27—C28—N1—Cu2	9.9 (2)
C3—C2—C7—C6	−1.8 (3)	O1—Cu2—N1—C17	−7.31 (18)
C1—C2—C7—C6	174.98 (16)	N2—Cu2—N1—C17	179.07 (19)
C5—C6—C8—O4	168.3 (2)	O5—Cu2—N1—C17	84.24 (18)
C7—C6—C8—O4	−13.9 (3)	O1—Cu2—N1—C28	163.50 (13)
C5—C6—C8—O3	−11.0 (3)	N2—Cu2—N1—C28	−10.12 (13)
C7—C6—C8—O3	166.82 (17)	O5—Cu2—N1—C28	−104.95 (13)
O5—C9—C10—C15	171.84 (17)	C25—C26—N2—C27	−0.5 (3)
O6—C9—C10—C15	−6.6 (3)	C25—C26—N2—Cu2	−174.86 (16)
O5—C9—C10—C11	−3.4 (3)	C23—C27—N2—C26	−1.9 (3)
O6—C9—C10—C11	178.12 (18)	C28—C27—N2—C26	178.76 (19)
C15—C10—C11—C12	−0.1 (3)	C23—C27—N2—Cu2	173.43 (15)
C9—C10—C11—C12	175.2 (2)	C28—C27—N2—Cu2	−5.9 (2)
C10—C11—C12—C13	−0.7 (4)	O3^ii^—Cu2—N2—C26	8.8 (2)
C11—C12—C13—C14	0.3 (4)	O1—Cu2—N2—C26	150.2 (3)
C12—C13—C14—C15	0.8 (3)	N1—Cu2—N2—C26	−176.6 (2)
C12—C13—C14—C16	−178.7 (2)	O5—Cu2—N2—C26	−78.90 (19)
C13—C14—C15—C10	−1.6 (3)	O3^ii^—Cu2—N2—C27	−165.84 (13)
C16—C14—C15—C10	177.98 (17)	O1—Cu2—N2—C27	−24.5 (4)
C11—C10—C15—C14	1.2 (3)	N1—Cu2—N2—C27	8.70 (13)
C9—C10—C15—C14	−174.09 (17)	O5—Cu2—N2—C27	106.44 (13)
C15—C14—C16—O8	7.6 (3)	O2—C1—O1—Cu2	−100.2 (2)
C13—C14—C16—O8	−172.9 (2)	C2—C1—O1—Cu2	81.5 (2)
C15—C14—C16—O7	−174.78 (18)	O3^ii^—Cu2—O1—C1	−16.66 (17)
C13—C14—C16—O7	4.8 (3)	N2—Cu2—O1—C1	−158.3 (3)
N1—C17—C18—C19	−0.1 (4)	N1—Cu2—O1—C1	168.91 (17)
C17—C18—C19—C20	1.9 (4)	O5—Cu2—O1—C1	70.49 (17)
C18—C19—C20—C28	−1.4 (3)	O1—C1—O2—Cu1	4.6 (2)
C18—C19—C20—C21	177.7 (2)	C2—C1—O2—Cu1	−177.06 (13)
C19—C20—C21—C22	−177.6 (2)	O6^i^—Cu1—O2—C1	−76.87 (13)
C28—C20—C21—C22	1.5 (3)	O6—Cu1—O2—C1	103.13 (13)
C20—C21—C22—C23	−1.6 (4)	O4—C8—O3—Cu2^ii^	21.2 (3)
C21—C22—C23—C27	−0.6 (3)	C6—C8—O3—Cu2^ii^	−159.55 (13)
C21—C22—C23—C24	−179.8 (2)	O6—C9—O5—Cu2	87.4 (2)
C27—C23—C24—C25	−1.3 (3)	C10—C9—O5—Cu2	−91.0 (2)
C22—C23—C24—C25	178.0 (2)	O3^ii^—Cu2—O5—C9	34.20 (17)
C23—C24—C25—C26	−0.8 (4)	O1—Cu2—O5—C9	−58.37 (17)
C24—C25—C26—N2	1.8 (4)	N2—Cu2—O5—C9	130.45 (17)
C24—C23—C27—N2	2.8 (3)	N1—Cu2—O5—C9	−146.85 (17)
C22—C23—C27—N2	−176.6 (2)	O5—C9—O6—Cu1	−10.3 (2)
C24—C23—C27—C28	−177.90 (19)	C10—C9—O6—Cu1	168.13 (12)
C22—C23—C27—C28	2.8 (3)	O2^i^—Cu1—O6—C9	84.72 (13)
C19—C20—C28—N1	−0.8 (3)	O2—Cu1—O6—C9	−95.28 (13)
C21—C20—C28—N1	179.97 (19)		

Symmetry codes: (i) −*x*+1, −*y*, −*z*+1; (ii) −*x*+1, −*y*, −*z*+2.

### Hydrogen-bond geometry (Å, °)

**Table:**

*D*—H···*A*	*D*—H	H···*A*	*D*···*A*	*D*—H···*A*
O7—H7A···O4^iii^	0.82	1.73	2.539 (2)	170

Symmetry codes: (iii) −*x*, −*y*, −*z*+2.
